# EHMTI-0350. Introducing a novel six-phase method for effective screening of the patients diagnosed with neurological electromagnetic hypersensitivity (EHS)

**DOI:** 10.1186/1129-2377-15-S1-L3

**Published:** 2014-09-18

**Authors:** M Khademi, SAR Mortazavi, M Haghani, SMJ Mortazavi

**Affiliations:** 1 School of Medicine, Shiraz University of Medical Sciences, Shiraz, Iran; 2 Ionizing and Non-ionizing Radiation Protection Research Center (INIRPRC), Shiraz University of Medical Sciences, Shiraz, Iran; 3 Ionizing and Non-ionizing Radiation Protection Research Center (INIRPRC), Shiraz University of Medical Sciences, Shiraz, Iran

## Background

Electromagnetic hypersensitivity (EHS) is a relatively new phenomenon characterized by experiencing neurological symptoms such as headache, dizziness, anxiety, and insomnia after exposure to electromagnetic fields (EMFs) like those generated by mobile phones and visual display units (VDUs). Although some published reports suggest that EHS is unrelated to the presence of electromagnetic fields, the etiology of EHS is not clearly known. Over the past years our lab has been studying the health effects of exposure of laboratory animals and humans to different sources of electromagnetic fields such as mobile phones, mobile base stations, mobile phone jammers, laptop computers, radars, dentistry cavitrons and MRI.

## Aim

In this paper we introduce a novel method for effective screening of the patients diagnosed with EHS.

## Method

We used a calibrated ICU monitoring system for recording the patient’s biological parameters such as systolic and diastolic blood pressure, mean arterial pressure, oral and peripheral temperature heart beat and respiration. Our method is composed of six consecutive phases; I: patient was exposed to mobile phone microwave radiations for 10 minutes. II: sham exposure for 10 minutes. III: same as phase I but the patient was informed that the mobile phone was on (in the talk mode). Phase IV, the patient was sham exposed and was informed that the mobile phone was switched off. V: same as phase I but the patient was given incorrect information (he/she was told that the mobile phone was switched off). VI: same as phase II but the patient was given incorrect information (he was told that the mobile phone was on). In each phase, patients were asked if they can feel the presence of electromagnetic fields.

## Results

Our preliminary results show that EHS patients cannot recognize real and sham exposures in different phases of the experiment. Furthermore, monitoring of the patient’s biological parameters in each phase could not show statistically significant differences between the means of biological parameters in real exposure and sham exposure phases.

## Conclusion

Altogether our preliminary findings confirm that under blind conditions, exposure to EMFs cannot trigger any subjective symptoms. It can be hypothesized that neurological symptoms such as headache in EHS patients is not caused by the presence of electromagnetic fields and lead us to this conclusion that psychological factors possibly play an important role in electromagnetic hypersensitivity.

No conflict of interest.

